# Hydatid Cyst of the Lung in Children: A Diagnosis Not to Be Missed

**Published:** 2019-04

**Authors:** Ahmed Khalil BEN ABDALLAH, Mohamed ZOUARI, Manel HAJ MANSOUR, Imen ABID, Mahdi BEN DHAOU, Mohamed JALLOULI, Riadh MHIRI

**Affiliations:** 1. Department of Pediatric Surgery, Hedi-Chaker Hospital 3029 Sfax, Tunisia; 2. Sfax Medical School, Sfax, Tunisia

## Dear Editor-in-Chief

Hydatid disease is an important public health problem in many Mediterranean and Middle East countries (1). “This disease is endemic in Tunisia. The annual incidence of chirurgical hydatid disease is 15/100000 in the Tunisian population” (2). The lungs are the most common sites of infection in children (3). The initial infection, as well as the development of a cyst, remains asymptomatic for many years. In some patients, symptoms such as dyspnea, coughing, and nausea and vomiting related to pressure may occur (4). The diagnosis is may be made incidentally on chest radiographs or CT scans (4). Surgery is the preferred treatment. Sole medical treatment is reserved for disseminated disease and inoperable patients (5).

The aim of the present study was to identify the epidemiological, diagnostic, and therapeutic features of pulmonary hydatidosis in children. Thirty patients under the age of 14 yr, operated between Jan 2009 and Dec 2016 due to pulmonary hydatid cysts, were evaluated retrospectively. All patients had chest radiographies and abdominal ultrasonography scans. Computed tomography scan was performed in 11 (36.6%) patients.

Written informed consent was obtained from the legal guardian of each patient to publish the case and accompanying images in scientific journals for research and educational purposes.

**Fig. 1:**
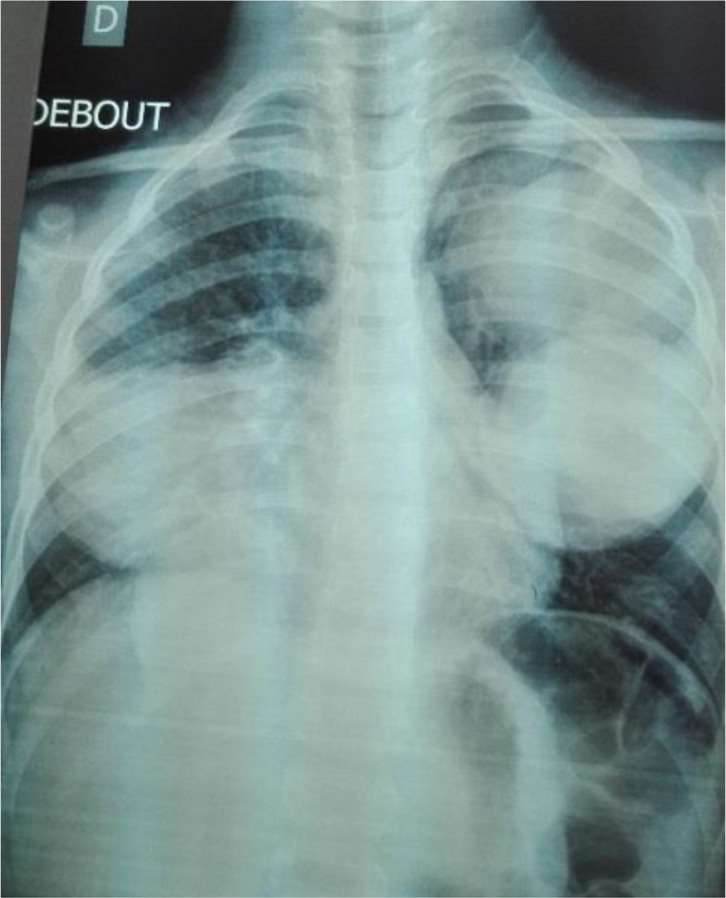
Radiography shows multiple hydatid cysts of the lungs, one in the right side and two in the left side

There were 21 boys and 9 girls. The mean age of our patients was 8.12 ± 2.5 yr. Twenty-eight (93.3%) patients were of rural origin. The symptoms at the time of presentation were coughing in 30% (n=9), hemoptysis in 20% (n= 6), and chest pain in 40% (n=12). The diagnosis was made incidentally on chest radiography in 10% (n=3). The average cyst diameter was 6.36 ± 1.8 (3–10) cm. The cysts involved the left lung in 16 (63.6%) patients, the right in 11 (36.4%). Three (10%) patients had bilateral lung cysts (Fig. 1).

**Fig. 2:**
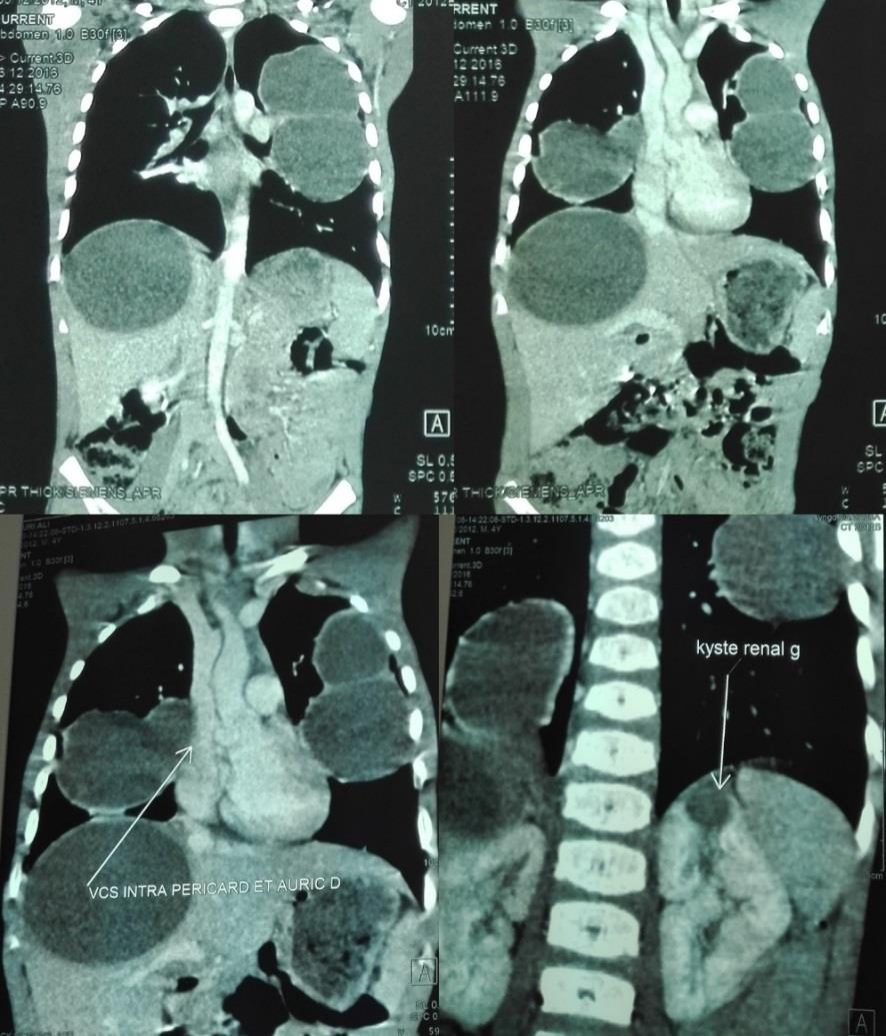
Chest and abdominal computed tomography images showing multiple cysts with involvement of the lungs, the liver and the left kidney

The most common location of cysts was in the left lower lobe (40%, n=12). Serological tests were positive in 22 (73.3%) patients. Twelve (40%) patients had perforated cysts. A liver hydatid cyst was found in 3 (10%) patients. One patient had multiple cysts with involvement of the lungs, the liver and the left kidney (Fig. 2). All of the patients were managed surgically. Posterolateral thoracotomy and cyst excision were performed in all patients. Three liver cysts were >5 cm and were managed with the puncture, aspiration, injection, and reaspiration (PAIR) technique. The lung was treated first in patients with concomitant lung and liver involvement. A second operation was performed for the liver within 2 to 4 months after the lung operation. In all patients, Albendazole was given perioperatively at a dosage of 10 mg/kg per day and was continued postoperatively for 2 months. The average follow-up period was 32.5 ± 23.3 months. No allergic reactions were observed, and there were no recurrences, owing to the preoperative planning and meticulous perioperative precautions to prevent spillage.

Although hydatid disease has been controlled in many countries, this zoonosis remains endemic in some regions, especially in developing countries. A high index of suspicion is needed to make the diagnosis. During surgical removal of hydatid cysts, we must avoid spillage of cyst contents to prevent anaphylactic reaction, recurrence and multiple hydatidosis. Prophylactic Community-based measures are crucial to treatment strategy. These measures include people education, treatment and control of all dogs, and proper hand washing after contact with dogs or their feces.
